# Effects of vitamin B12 supplementation on oxidative stress markers and pro-inflammatory cytokines during pregnancy and postpartum among Bangladeshi mother–child pairs

**DOI:** 10.1186/s40795-023-00785-y

**Published:** 2024-01-03

**Authors:** Towfida Jahan Siddiqua, Evana Akhtar, Md. Ahsanul Haq, Seterah Shahab-Ferdows, Daniela Hampel, Sharmin Islam, Tahmeed Ahmed, Lindsay H. Allen, Rubhana Raqib

**Affiliations:** 1 International Centre for Diarrheal Disease Research, Dhaka, Bangladesh; 2 USDA ARS Western Human Nutrition Research Center, Davis, CA USA; 3 Department of Nutrition, University of California, Davis, CA USA

**Keywords:** Vitamin B12, Lactation, Human milk, Oxidative stress,cytokines

## Abstract

**Background:**

There is limited research to determine whether vitamin B12 (B12) supplementation during pregnancy and lactation is protective against oxidative stress and pro-inflammatory cytokines and whether this effect is transferred to breastfed infants via milk. In addition, associations among maternal plasma/ milk and infant B12 status and immune function markers are poorly characterized.

**Objectives:**

To evaluate effects of oral B12 supplementation during pregnancy and postpartum on maternal and infant 8-hydroxy-2′-deoxyguanosine (8-OH-dG, an oxidative stress marker) and proinflammatory cytokine levels, and examine associations between maternal plasma, breastmilk and infant B12 status as well as immune function markers.

**Method:**

In a blinded, placebo-controlled trial, Bangladeshi women (*n* = 68, 18–35 years, hemoglobin < 11 g/dL, gestational weeks 11–14) received either 250 μg/day B12 or placebo throughout pregnancy up to 3-months postpartum. Samples were collected from mothers at baseline and 3-months postpartum and from infants at 3-months to measure B12 status indicators, 8-OH-dG and proinflammatory cytokines.

**Results:**

Maternal postpartum B12 was positively associated with infant plasma B12. Higher milk B12 concentrations were associated with increased infant B12 (beta (β) = 277, 95% confidence interval (CI) = (132, 423), *p*<0.001) and lower total homocysteine (β = -7.63, 95% CI = (-12.40, -2.86), *p* = 0.002) levels. Maternal B12 supplementation reduced plasma 8-OH-dG concentrations among postpartum mothers and infants compared to the placebo group. Supplementation increased plasma TNF-α and IL-6 levels among mothers and IL-10 and IFN-γ levels among infants.

**Conclusion:**

Milk and maternal plasma B12 at 3 months were associated with infant B12. Maternal B12 supplementation modulates 8-OH-dG and several cytokines which may protect against immune response-induced oxidative stress.

**Trial registration:**

(clinicaltrials.gov: NCT01795131- 1^st^ posted on 20/02/2013).

**Supplementary Information:**

The online version contains supplementary material available at 10.1186/s40795-023-00785-y.

## Introduction

Vitamin B12 (B12) is a water-soluble essential micronutrient which is mainly obtained from animal-sourced food (including meat, fish, eggs, and dairy) [1] with critical role in DNA, protein, and lipid synthesis. Vitamin B12 serves as a cofactor for the enzyme methionine synthase and enzyme methyl-malonyl-CoA (MMA) mutase that catalyze the conversion of homocysteine into methionine and the conversion of methyl-malonyl-CoA to succinyl-CoA respectively. Vitamin B12 deficiency leads to accumulation of total homocysteine (tHcy) and methyl malonic acid (MMA) impairing DNA synthesis and resulting hematologic abnormalities [2]. Globally, B12 insufficiency is most prevalent in women and children, especially in resource-poor regions where the consumption of animal-source food is limited [1, 3, 4]. Several studies have demonstrated that B12 deficiency or limited B12 intake during pregnancy are associated with adverse outcomes, including intrauterine growth restriction, low birth weight, neural tube defects and preterm delivery [5, 6]. Children born to B12-deficient mothers have low B12 stores at birth, which may be further exacerbated by a very low concentration of B12 in breastmilk, and may hinder the children’s growth and development [7]. However, the extent to which maternal serum/plasma B12 status during pregnancy and breastmilk B12 concentrations determine the B12 status of infants remains controversial [8–11].

The consequences of mild or subclinical B12 deficiency (serum concentration 87-148 pg/mL) are not well-characterized, but have been associated with enhanced inflammatory oxidative stress [12]. Moreover, several aspects of cell-mediated and humoral immunity can be compromised by B12 deficiency, thus B12 is hypothesized to function as an important anti-inflammatory agent [13]. Cytokines are critical regulators of the immune response and inflammation, and B12 can modulate cytokines to confer protection against immune response-induced oxidative stress, which can trigger the release of pro-inflammatory factors [12, 14]. Current evidence indicates that B12-deficient adipocytes increase the gene expression of pro-inflammatory cytokines (such as interleukin-1 (IL-1) interleukin-6 (IL-6), interleukin-8 (IL-8), interleukin-18 (IL-18), transforming growth factor beta (TGF-β), tumor necrosis factor alpha (TNF-α), monocyte chemoattractant protein-1 (MCP-1/CCL2)) [15]. Tamura et al. demonstrated that methyl-B12 treatment can augment both CD8^+^T lymphocytes and natural killer (NK) cell activity in patients with B12 deficiency, suggesting an important role of B12 in regulation of the immune system, though the precise mechanisms are unclear [16]. Few studies have evaluated the association between B12 status, or the effects of B12 supplementation or interventions, on immune response [17, 18].

Furthermore, B12 may also function as a scavenger of reactive oxygen species [12, 19]. A study among patients with diabetes reported a significant association between B12 status and oxidized low-density lipoprotein-cholesterol, particularly among patients adhering to a vegetarian diet [20], B12 supplementation may prevent cellular damage in patients with genetic disorders of B12 metabolism [19]. However, there is limited information available as to whether circulating B12 concentrations are related to inflammatory oxidative stress. Early infancy is a vulnerable period of development and provision of prenatal B12 supplements may improve immune function. We previously reported that maternal B12 supplementation during pregnancy through 3 months postpartum reduced the proportion of infants with elevated levels of alpha-1-acid glycoprotein (AGP) and C-reactive protein (CRP), which suggests that B12 might alleviate inflammatory responses in infants [21]. Growth (IL-2) and effector (IL-4, IL-6, IL-10, IFN-γ, and TNF-α) cytokines are critical components of effective adaptive immunity [22]. To the best of our knowledge, no studies have employed measures of immune function or T-cell responses as an indicator to evaluate the effects of maternal B12 supplemental dose on the immune function of infants. Moreover, there is a lack of research on the relationships among maternal plasma, milk, infant B12 status and the multiple immune function markers mentioned above in a population group at risk of deficiency, e.g. women of low-socioeconomic status.

Thus, in this study we aimed to evaluate the beneficial effect of oral B12 supplementation during pregnancy and postpartum reducing maternal and infant concentrations of oxidative stress biomarker, 8-hydroxy-2-deoxyguanosine (8-OHdG), one of the primary downstream products of DNA oxidation. Plasma 8-OHdG is considered the as gold standard biomarker for estimating the oxidative DNA damage; moreover it is more stable than the other oxidative stress markers and earlier report demonstrated that chromosomal and oxidative DNA damage condition can be prevented by pretreatment with B12 (by oxidative DNA damage) [23]. We also compared the effects of supplementation on a panel of cytokines (including IL-2, IL-4, IL- 6, IL-10, IFN-γ, TNF-α) that are generally known to play a role in the Th1/Th2 balance. Furthermore, we assessed the associations between biomarkers of B12 status (by measuring plasma methylmalonic acid, total homocysteine), B12 concentrations in milk and immune markers, among the mother-infant pairs. This study was nested within a randomized controlled trial of B12 supplementation during pregnancy and lactation conducted in Bangladesh.

## Materials and methods

### Study design and participants

A blinded, placebo-controlled trial was carried out by the Maternal and Child Health Training Institute (MCHTI), Azimpur, Dhaka, Bangladesh, a non-governmental facility that provides basic antenatal and obstetric services to a low-income community. Briefly, pregnant women residing in the communities of Azimpur and Kamrangir Char in the neighboring areas of MCHTI were enrolled and followed-up in a clinical trial of B12 supplementation. The inclusion criteria were: women aged 18 to 35 years currently residing in Dhaka, during 11–14 weeks of gestation, and hemoglobin within 7–11 g/dL who planned to deliver at the MCHTI, and stay in Dhaka throughout their pregnancy and for up to three months post-delivery. The exclusion criteria were: women with severe anemia (hemoglobin < 7 g/dL), having the history of previous complicated pregnancies or of pre-term delivery, abortion; presence of any systemic disease and had received influenza vaccine. Study participants were randomized to receive either 250 μg of B12 (methyl cobalamin) per day or a placebo starting at early pregnancy continued through 3 months postpartum. Both groups also received 400 µg folic acid and 60 mg Fe (iron–folic acid) as part of the routine care of pregnant women. The women were also immunized with an influenza vaccine (H1N1) at 26 to 28 weeks of gestation [21].

This study was reviewed and approved by the Research Review Committee (RRC) and Ethical Review Committee (ERC) of the International Centre for Diarrhoeal Disease Research, Bangladesh (icddr,b), and the Human Research Protection Office of the University of California, Davis, CA, USA. This intervention trial was registered in the clinical trial registry (clinicaltrials.gov: NCT01795131). Written informed consent was obtained from all eligible participants. The data collected from each participant included maternal height and body weight, gestational age at delivery, birth weight, compliance with supplementation, and consumption of animal source foods (ASF) rich in B12 (meat, egg, fish, dairy products) was recorded by a dietary diversity questionnaire during the previous 7 days.

### Sample collection and biochemical measurements

Peripheral blood samples (*n*= 68) were collected from the mothers at enrollment and 3 months postpartum, and from infants at the age of 3 months. Colostrum (5 mL) was collected within 72 h of delivery usually at morning and breastmilk samples (5 mL) were obtained at 3 months postpartum as previously described [21].

The separated EDTA plasma samples were stored at -80 °C for analysis at the icddr,b, unless indicated otherwise. Total Hb in whole blood (500 μL) was quantified by Drabkin's cyanmethemoglobin method [24]. Each sample was analyzed in duplicate and absorbance at 542 nm was measured by spectrophotometry (SIGMA, St. Louis, MO, USA). The inflammation biomarker, alpha-1-acid glycoprotein (AGP) was determined in plasma by an immunoturbidimetric assay using a commercial kit (Roche diagnostics GmbH, Mannheim, Germany) on a Roche automated clinical chemistry analyzer (Hitachi-902, Boehringer Mannheim, Germany). Plasma Hs-CRP was determined using a Cobas Integra® 400 plus Analyzer (Roche Diagnostics). Plasma folate and B12 were measured using an electrochemiluminescence immunoassay on a Cobas e6000 using a commercially available kit (Roche Diagnostics GmbH, Mannheim, Germany). Plasma concentrations of oxidative stress marker, 8-OH-dG were assessed using a commercial ELISA kit (Japan Institute for the Control of Aging, JaICA) and cytokines (e.g., IL-2, IL-4, IL- 6, IL-10, IFN-γ, TNF-α) mostly pro-inflammatory cytokines were measured using BD™ Cytometric Bead Arrays (CBA) Kit (BD Biosciences, San Jose, CA, USA).

Plasma methylmalonic acid (MMA) concentrations were analyzed by liquid chromatography-tandem mass-spectrometry (UPLC-MS/MS) [25] and plasma total homocysteine (tHcy) was determined by high-performance liquid chromatography with fluorescence detection (HPLC-FLD; Agilent 1200; Burnsville, MN, USA) at the USDA/ARS, Western Human Nutrition Research Center (WHNRC) at the University of California, Davis, CA, USA [26]. Colostrum and breastmilk B12 analysis was carried out at the WHNRC on a Siemens IMMULITE® automated, quantitative immunoassay analyzer (Duluth, GA) [27].

### Sample size

Current study was nested in a parent trial, where a total of 82 pregnant women were enrolled; among them sixty-eight mother and infant pairs (35 in the placebo group and 33 in the B12 group) completed the ~ nine-month follow-up. Loss to follow-up was mostly due to migration out of the study area (*n* = 5), being unreachable (*n* = 3), refusal to take the capsule (*n* = 2) and miscarriage (*n* = 4) [21]. Due to data unavailability regarding maternal vitamin B12 supplementation on maternal or infant inflammatory oxidative stress responses, in this study, sample size was calculated considering the allowable arbitrary mean difference in oxidative stress response between the supplemented and placebo group would be 75% of the population standard. Thus, the estimated sample size based on t-test was 30 in each group at α = 0.05 and 80% statistical power. Unused/intact plasma samples were available from 63 maternal and infant pairs (30 from supplemented and 33 from placebo group). Thus, in total 63 mother-infant pairs were considered in the current study.

### Statistical methods

Statistical analyses were conducted using IBM SPSS Statistics for Windows (version 20; Armonk, NY: IBM SPSS Corp.; 2011) and Stata software (Release 14; StataCorp LP, College Station, TX 77845, USA). Histograms, bar diagrams and pie charts were used for data visualization, as appropriate. Descriptive statistics such as proportions, mean and standard deviations for normally distributed data and medians and inter‐quartile ranges (IQR) for non-normally distributed quantitative variables were used to summarize the data. The Chi-square test was used to assess the associations between two categorical variables and *t*-tests were used to examine the significance of the differences between the two groups for normally distributed variables. Two-way repeated measures ANOVA was used to estimate the significance of the differences in the plasma 8-OH-dG and cytokine levels between baseline and 3 months postpartum within each group.

In order to determine normality graphically, we used the output of a normal Q-Q Plot. Simple linear regression and Pearson's correlation were primarily used to investigate the bi-variate associations between the outcomes and other variables. To observe the difference in plasma 8-OH-dG and cytokine concentrations (IL-2, IL-4, IL- 6, IL-10, IFN-γ, TNF-α) between B12 supplementation and placebo groups, multivariate regression model was used. The regression models were adjusted for maternal age and BMI, the sex of the child, birth weight, and mode of delivery. Further adjustment was performed for the baseline outcome values when significant differences were observed at baseline. *P*-values < 0.05 were considered significant.

## Results

### Demographic characteristics

The demographic characteristics of the mother–child pairs did not differ significantly between groups at baseline. The mean age of the women at enrollment was 22.5 years and the mean BMI was within the normal range (< 25.0 kg/m^2^). C-section births accounted for one-fourth of all births, and 50.7% of infants were male. No children were excluded due to premature birth (Table 1).

**Table 1:** Baseline demographics of the mother–child pairs

Characteristic	All	B12 group	Placebo	*p*-value
**Pregnant women**				
Age of women (years)	22.5 ± 3.6	22.6 ± 3.34	22.3 ± 3.86	0.691
BMI at GW8 (kg/m^2^)	21.2 ± 4	21.6 ± 1.91	22.1 ± 1.74	0.221
Delivery mode				
Vaginal	54 (74.0%)	24(68.6%)	30(78.9%)	0.229
Cesarian	19 (26.0%)	11(31.4%)	8(21.1%)	
Gestational age, GW (weeks)	38.8 ± 1.8	38.2 ± 1.90	38.6 ± 1.62	0.388
Hb (g/dL)	11.2 ± 0.92	11.2 ± 0.86	11.2 ± 1.00	0.867
Folate, nmol/L	8.3 ± 4.1	8.99 ± 4.81	7.74 ± 3.45	0.213
B12, pg/mL	139.2 (109.7, 176.43)	142.3(109.1, 163.8)	134.7(110.5, 201.7)	0.714
tHcy, µmol/L	9.2 (8.0, 11.0)	9.30(8.20, 11.5)	9.00(7.90, 10.5)	0.314
MMA, nmol/L	220.0 (168.0, 324.0)	228.0(158.0, 317.0)	219.5(148.0, 318.5)	
**Children**				
Birth weight (g)	2,786 ± 385	2,776 ± 338	2,796 ± 410	0.811
Boys	37 (50.7%)	18(51.4%)	19(50.0%)	0.545
Girls	36 (49.3%)	17(48.6%)	19(50.0%)	

Data are expressed as mean ± SD or median with inter quartile range (IQR) or numbers with percentages in parenthesis. Independent sample t-test for normalize data and non-parametric Mann–Whitney U test or chi-square test was used for non-normalize data

*BMI* Body mass index, *Hb* Hemoglobin, *GW* Gestational age, *tHcy* total homocysteine, *MMA* Methylmalonic Acid

At three months post-partum, the proportion of anemia was slightly higher for infants (26.0%) than mothers (16.4%; *p* = 0.775). Following supplementation, the plasma and milk B12 concentrations of the supplemented group increased significantly (Supplementary Table 1). As expected, the estimated B12 status markers of infants born to supplemented mothers showed significantly better status than those of infants the placebo group (Supplementary Table 1).

### Comparison of 8-OH-dG and cytokine levels at 3 months postpartum

Two-way repeated measures ANOVA revealed that the plasma concentration of 8-OH-dG decreased in both groups between baseline and 3-months postpartum. However, this difference was only significant in the supplemented group (Diff = -1.18 (-2.19, -0.17*; p* = 0.023) and not in the placebo group (*p* = 0.376) (Table 2). Multivariate regression showed that B12 supplementation significantly reduced the 8-OH-dG concentrations among both the mothers (*p* = 0.002) and infants (*p* = 0.001) at 3 months compared to the placebo group (Table 3).

**Table 2:** Within group changes in plasma 8-hydroxy-2′-deoxyguanosine (8-OH-dG) and cytokines between baseline and 3 months post-partum

	Placebo (*n* = 33)	*P*-value	B12-group (*n* = 30)	*P*-value
^a^8-OHdG	-0.38 (-1.23, 0.48)	0.376	-1.18 (-2.19, -0.17)	0.023
^a^IFN-γ	-0.02 (-0.83, 0.79)	0.962	-0.52 (-1.83, 0.79)	0.423
TNF-α	-0.11(-0.56, 0.35)	0.629	-16.7 (-51.5, 18.1)	0.334
^a^IL-10	0.24 (-0.56, 1.04)	0.543	-0.49 (-1.07, 0.09)	0.096
IL-6	-1.08 (-2.50, 0.33)	0.129	-27.8 (-85.1, 29.5)	0.329
IL-4	0.08 (-0.41, 0.57)	0.741	0.73 (-0.76, 2.21)	0.323
^a^IL-2	0.08 (-0.24, 0.40)	0.612	0.25 (-0.46, 0.96)	0.478

Data were presented as beta coefficient with 95% confidence interval within parenthesis. Two-way repeated measures ANOVA was used to estimate the *P*-values and the model was adjusted by mother’s age, BMI, mode of delivery; and infants’ birth weight and sex

^a^Data were log transformed

**Table 3:** Plasma 8-hydroxy-2′-deoxyguanosine (8-OH-dG) and cytokine concentrations of the participants from placebo and B12 supplementation group

Variables	Placebo (*n* = 33)	B12-group (*n* = 30)	*p*-value
**8-OHdG (ng/mL)**			
Baseline	6.13 ± 1.85	4.99 ± 2.61	0.056
3 months postpartum (mother)	5.75 ± 3.01	3.81 ± 1.48	0.002
3 month (Infant)	5.41 ± 2.59	3.57 ± 1.57	0.001
**IFN-γ (pg/mL)**			
Baseline	4.12 ± 2.19	6.26 ± 4.32	0.017
^a^3 months postpartum (mother)	4.10 ± 2.41	5.74 ± 3.99	0.602
3 month (Infant)	4.73 ± 2.86	9.43 ± 6.20	< 0.001
**TNF-α (pg/mL)**			
Baseline	2.22 ± 1.03	3.18 ± 1.65	0.296
3 months postpartum (mother)	2.11 ± 1.01	3.44 ± 3.56	0.043
3 months (Infant)	2.58 ± 1.29	3.31 ± 2.05	0.086
**IL-10 (pg/mL)**			
Baseline	2.74 ± 0.77	3.70 ± 1.74	0.004
^a^3 months postpartum (mother)	2.98 ± 2.25	3.19 ± 1.25	0.413
3 months (Infant)	6.14 ± 2.93	7.63 ± 3.02	0.041
**IL-6 (pg/mL)**			
Baseline	5.49 ± 3.29	5.71 ± 2.68	0.321
3 months postpartum (mother)	4.36 ± 2.31	5.90 ± 2.23	0.011
3 months (Infant)	9.24 ± 8.33	9.44 ± 9.29	0.333
**IL-4 (pg/mL)**			
Baseline	2.01 ± 1.13	2.53 ± 1.48	0.118
3 months postpartum (mother)	2.09 ± 1.10	3.25 ± 4.08	0.118
3 months (Infant)	2.02 ± 1.11	2.48 ± 1.54	0.150
**IL-2 (pg/mL)**			
Baseline	1.79 ± 0.83	2.31 ± 0.99	0.024
^a^3 months postpartum (mother)	1.87 ± 0.73	2.56 ± 2.35	0.642
3 months (Infant)	1.98 ± 0.61	2.25 ± 0.81	0.150

Data are presented as mean ± standard deviation (SD). Multivariate regression model was used to estimate the *p*-value and model was adjusted by mother’s age, BMI, mode of delivery; and infants’ birth weight and sex

^a^Adjusted by baseline outcomes those were differed significantly at baseline

The levels of three (IFN-γ, TNF-α, IL-6) of the six cytokines examined decreased in both groups between baseline and 3-months postpartum; however, these differences were not significant (Table 2). IL-10 is produced both by Th2 cells and regulatory T cells. Interestingly, the reduction in IL-10 showed a tendency towards significance in the B12-supplemented mothers (*p* = 0.096). In this study, B12 supplementation did not seem to influence the cytokine concentrations within group over time significantly (Table 2).

Supplementation significantly increased maternal levels of TNF-α and IL-6 (*p* = 0.043 and *p* = 0.011) compared to the placebo group (Table 3) after 3 months of postpartum. Compared to the infants in the placebo group, the infants born to the B12 supplemented mothers exhibited significantly higher plasma IFN-γ and IL-10 concentrations (*p* < 0.001 and *p* = 0.041) (Table 3). However, supplementation with B12 from gestational week (GW) 14 to 3-month post-partum did not lead to significant alterations in maternal levels of IFN-γ, IL-2 or IL-4 compared to the placebo group, or in other cytokines (TNF-α, IL-2, IL-6) in infants.

### Predictors of infant B12 status

**Fig. 1:**
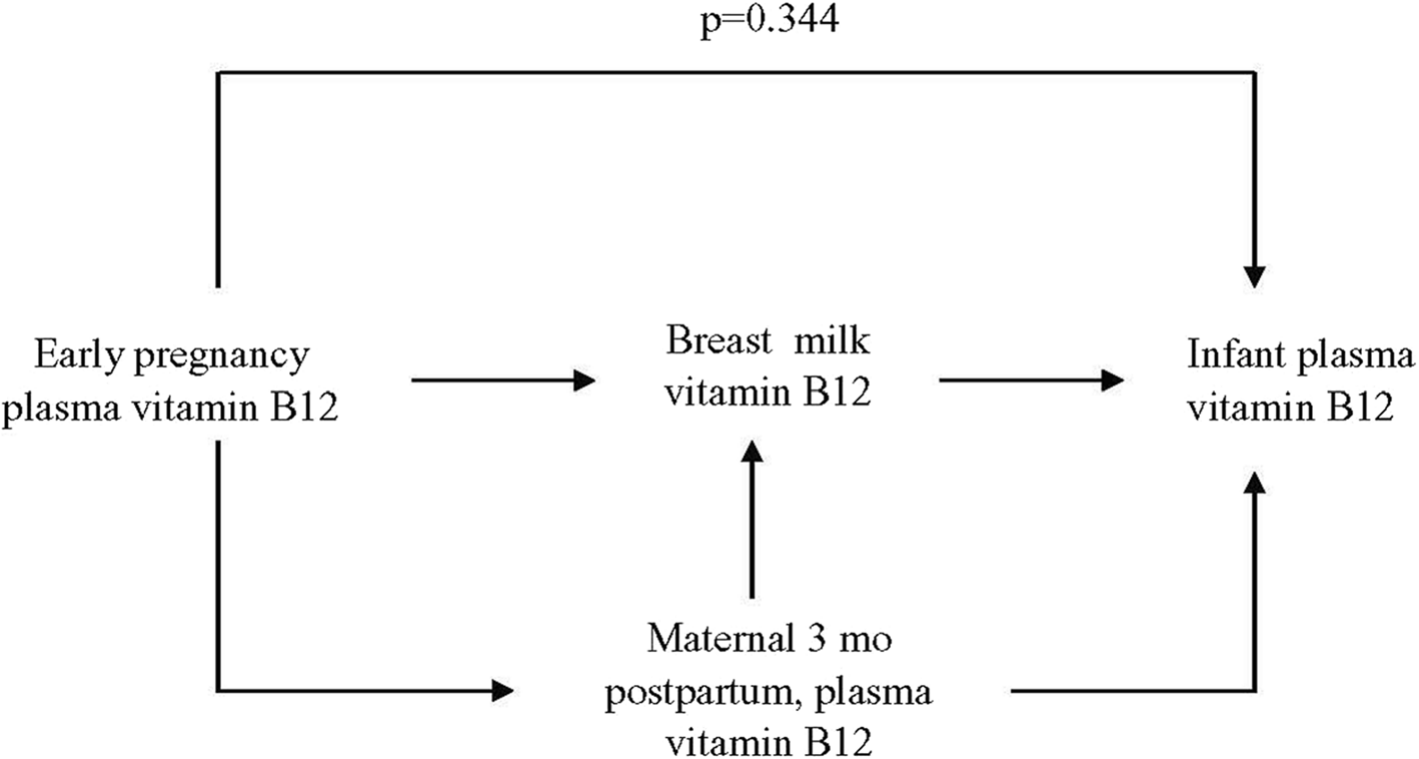
Best model for predicting plasma vitamin B12 concentrations in 3-month-old Bangladeshi infants (*n* = 68, all *p* < 0.05). A multivariate regression model adjusted for the mother’s age, BMI, mode of delivery; and birth weight and the sex of the child, was used to estimate the *p*-values

To investigate the relative influence of various factors on infant B12 status, regression analyses were performed to establish a model to predict how maternal plasma B12 status affects the level of B12 in milk, and subsequently the plasma B12 levels of infants (Supplementary Table 2). Adjusted multivariate regression analysis showed the early pregnancy maternal plasma B12 concentrations were positively associated with breastmilk B12 concentrations at 3 months postpartum but not significantly associated with infant plasma B12 levels (*p* = 0.344) (Fig. 1). The breastmilk B12 concentrations at 3 months postpartum were significantly, positively associated with maternal plasma B12 concentrations during early pregnancy and at 3 months postpartum among both groups overall. Notably, the B12 concentrations in milk were associated with maternal plasma B12 levels only in the supplemented group (*p* = 0.002) at 3 months postpartum (Supplementary Table 2).

We did not observe any associations between milk B12 levels and maternal plasma MMA and tHcy concentrations at 3 months postpartum (Supplementary Table 2). Overall, milk B12 concentrations were positively associated with infant plasma B12 (β = 277, 95% CI = 132, 423, *p* < 0.001) and negatively with tHcy (β = -7.63, 95% CI = -12.40, -2.86, *p* = 0.002).

The colostrum B12 concentrations were significantly associated with infant plasma B12 (β = 19, 95% CI: 8, 230; *p* = 0.037), but not with infant plasma MMA and tHcy concentrations. The maternal plasma B12 concentrations at 3 months postpartum were associated with infant plasma B12 but not with infant MMA and tHcy. There were significant associations between maternal and infant tHcy and MMA.

### Associations between biomarkers of vitamin B12 status and 8-OH-dG and cytokine concentrations

Data from the placebo and supplemented groups were merged in the association studies in order to determine the association between biomarkers of B12 status and inflammatory oxidative stress markers. A multivariate regression model was established to investigate the associations between B12 status (B12, MMA, tHcy) of the mothers and infants and plasma biomarkers at baseline and 3-months postpartum. After controlling for covariates/confounders (mother’s age, mode of delivery, sex of the child), we observed a positive association between IFN-γ and plasma B12 (β = 6.56, 95% CI = -0.32, 13.4) among infants. Further, a significant, inverse association was also observed between infant tHcy and IFN-γ (β = -14.45, 95% CI = -22.67, -6.23) and MMA and IFN-γ (β = -5.14, 95% CI = -10.4, 0.15). On the other hand, no significant associations were observed between the B12 biomarkers and 8-OH-dG or any other cytokines (IL-2, IL-4, IL- 6, IL-10, TNF-α) among either mothers or infants.

### Associations between acute phase proteins and cytokines

We observed a significant, negative association between AGP and TNF-α among mothers (β = -6.48, 95% CI = -11.8, -1.14). In contrast, a positive association was observed between AGP and TNF-α among the infants at 3 months (β = 2.60, 95% CI = 0.25, 4.94), indicating the acute phase response of AGP correlates with the production of inflammatory cytokines in infants. No such associations were noted between AGP or CRP levels and the levels of other pro-inflammatory cytokines. We have previously shown B12 supplementation reduced AGP among infants, but no effect was observed in mothers.

## Discussion

Supplementation with 250 μg/day B12 throughout pregnancy and the first 3 months of lactation significantly reduced the plasma oxidative stress marker 8-OH-dG in both mothers and infants. We previously reported that supplementation increased B12 in plasma, colostrum and milk (*p*< 0.05) [22]. Here we further report of the influence of B12 supplementation on induction of both Th2 and Th1 cytokines. In addition, the data revealed that both milk B12 and maternal plasma B12 levels at 3 months were associated with the infants’ plasma B12 concentrations.

Our findings indicate the beneficial effects of B12 supplementation on oxidative stress, as the results of this study support the hypothesis that B12 supplementation may reduce oxidative stress in mothers and their infants. These findings are consistent with in vitro studies in cell-free systems, neuronal cells and human aortic endothelial cells, in which B12 supplementation significantly reduced the concentrations of reactive oxygen radicals, e.g. superoxide [19, 20]. Although maternal B12 supplementation did not raise the median B12 concentrations in milk to the assumed adequate level (310 pmol/L) [8], it did reduce infant B12 deficiency, as evidenced by their lower concentrations of methylmalonic acid and total homocysteine. We hypothesized that pregnant women would have a significantly lower B12 status compared to non-pregnant women, most likely due to pregnancy itself as well as increased requirements for the growing fetus, and consequently the resulting low B12 status of pregnant women may lead to increased oxidative stress and inflammation. Although the mechanisms remain poorly defined, the potential antioxidant properties of B12 include efficient and direct scavenging of reactive oxygen species (ROS), indirect induction of ROS scavenging by preservation of glutathione, and reduction of homocysteine-induced oxidative stress [6]. Our study also adds to the evidence base on the appropriate dosage and duration of B12 supplementation during pregnancy. A previous study in India [9] showed that 50 μg/d of cyanocobalamin throughout pregnancy and 6 weeks postpartum did not meaningfully improve the B12 status of mothers or infants, whereas our dose of 250 μg/day not only increased B12 status, but also reduced the oxidative stress marker. Thus, it is likely that 50 μg/day of B12 is not a sufficient amount to improve milk B12 concentrations, and therefore would not have any effects on stress and inflammation. However, in the Indian study the milk B12 concentration was quite low and the mothers were more B12-deficient, which could indicate that the effect on stress may not only be dependent on B12 dose, but also on maternal status itself, i.e., if the mother has such low stores of B12, the effect on oxidative stress may not be evident as the B12 is being utilized for its primary cellular functions. Additional trials are needed to explore the therapeutic potential of B12 against oxidative stress including evaluation of additional biomarkers of oxidative stress, ROS scavengers and anti-oxidants.

Our results demonstrated that two cytokines, TNF-α and IL-6, increased in response to vitamin B12 supplementation of pregnant women. Moreover, TNF-α and AGP concentrations were associated in both mothers and infants. Both TNF-α and IL-6 play important roles in the regulation of B cell function. Previous clinical and experimental observations revealed a novel role for B12 in modulation of the expression of cytokines through modifying the activity of transcription factors, such as nuclear factor-κB, a prototypical proinflammatory signaling pathway [28]. Interleukin-2, IFN-γ and TNF-α are the principal cytokines released by Th1 lymphocytes. TNF-α is a “master-regulator” of inflammatory cytokine production, while IL-6 acts as both a pro-inflammatory and an anti-inflammatory cytokine. Studies have reported that B12 deficiency leads to overproduction of TNF-α in B12-deficient rats and severely B12-deficient patients [29, 30]. Yamashiki et al. reported methyl B12 suppressed production of the pro-inflammatory cytokines IL-1 and IL-6 by peripheral mononuclear cells in vitro, reflecting Th1 suppression. [31] Moreover, induction by rIL-2 mitogens decreased production of the proinflammatory cytokine IFN-γ in a dose-dependent manner. A study of adults with Alzheimer revealed upregulation of IL-6 production in patients with low plasma B12 levels compared to patients with normal B12 levels [32].

We observed a significant increase in a Th1 cytokine (IFN-γ) among the infants of supplemented mothers compared to placebo group. Interestingly, a similar pattern was observed for the anti-inflammatory cytokine IL-10, which is produced by both Th2 cells and regulatory T cells. Since vitamin B12 plays a critical role to protect against low-grade inflammation-induced oxidative stress, these transient increases in cytokines in a balanced manner may beneficially coordinate the activity of Th1 cells, NK cells and macrophages, all of which are required for optimal pathogen clearance. Cytokine production, particularly in response to exogenous stimuli, was altered in the supplemented mothers and infants. The mothers in this study were immunized with H1N1 [21], which enabled us to evaluate the effect of B12 supplementation on adaptive response to a vaccine. We demonstrated that women who received B12 supplementation developed a significantly improved response to the influenza vaccine and maternal supplementation effectively reduced the proportion of children with an elevated acute phase response compared to the placebo [21]. In this study, a balanced response was seen where both Th1 and Th2 cytokines were generated without tilting towards a pro- or anti-inflammatory response. Both TNF- α and IFN-γ are Th1 (so called). TNF-α was higher in mothers while IFN- γ was higher in children compared to respective placebo. Only IL-10 (Th2) was high in infants (not mothers). It is noteworthy that both pregnancy and early postnatal life are chiefly viewed as Th2 phenomena.

Our analysis revealed that maternal plasma as well as milk B12 at 3 months postpartum were associated with infant plasma B12. Given that infants of well-nourished mothers are born with a store of ~ 25 µg of B12, it is now evident that maternal B12 status during pregnancy is a more superior indicator of infant B12 adequacy than milk B12 concentrations [33]. Previous studies have also reported positive correlations between maternal plasma/milk and infant’s serum/plasma total B12 concentrations [13, 26, 27]. For example, the Breastfeeding, Antiretroviral, and Nutrition (BAN) study reported a positive association between maternal and infant B12 concentrations at 2 or 6 weeks and at 24 weeks postpartum among Malawian mother–child dyads. The same study also reported significant correlations between maternal plasma, milk and infant plasma B12 concentrations at both 2 or 6 and 24 weeks. We observed similar patterns in our data, in that current maternal B12 status (as indicated by plasma and milk concentrations) was an important predictor of the infants’ B12 status at 3 months-of-age. Furthermore, colostrum B12 concentration were strongly positively associated with infant plasma B12. This supports previous studies that concluded that maternal B12 status could influence infants’ B12 status by increasing the infants’ B12 stores in utero as well as through amplified breastfeeding of infants with high B12 concentrations.

In this study, milk B12 concentrations were more strongly associated with infant B12 status than maternal plasma in utero*,*or colostrum. This observation raises the possibility that milk B12 could be a non-invasive B12 biomarker of maternal and/or infant B12 status. Current adequate dietary B12 intake is generally assumed to have beneficial effects for both mothers and their offspring’s, even if B12 status is poor before conception or during early pregnancy. The B12 concentration of human milk is affected by the mother’s dietary B12 intake and her stores [12]; however, the reported associations between milk and infant B12 concentrations are inconsistent. A longitudinal study of 25 Danish mother–child dyads observed a positive correlation between milk and infant plasma B12 concentrations at 4 months postpartum, but not at 2 weeks or 9 months [13]. A study of 113 Guatemalan dyads found milk B12 concentrations were inversely associated with infant B12 status (as assessed by urinary methyl malonic acid) at 3 months postpartum [15]. Based on previous methods that may have under- or over reported the amount of B12 in milk, concerns have been raised about the validity of breastmilk B12 values used to set Adequate Intakes for infants. However, to quantify B12 in milk, we used a validated commercial assay that is capable of assaying milk samples with very low B12 concentrations and does not require laborious pretreatment steps.

This study has several strengths, including the double-blind, placebo-controlled design and recruitment of a healthy and homogeneous group of subjects with low baseline B12 stores. One unique aspect of this study was the assessment of additional biomarkers namely MMA and tHcy, for mothers and infants to corroborate the interpretation of B12 deficiency, which enabled us to assess the associations between various biomarkers with the outcome measures, rather than relying solely on plasma B12. This study also provides some of the first data on the effects of B12 supplementation on inflammatory oxidative stress. The lack of longitudinal data is a limitation, especially given that vit B12 concentrations appear to fluctuate throughout lactation. Other shortcomings of the study include the relatively small sample size and the lack of data on dietary intake of B12, which limit our ability to generalize these findings to populations with high- or low-B12 intakes. One of the primary limitations of this study is the lack of data on the infectious disease incidence and other confounders of inflammatory status for the mother-infant pairs. Measurement of cytokines in culture supernatant of stimulated cells would have given a better indication of the functional status of the cells, instead of serum levels of cytokines. However, we could not measure cytokines in culture supernatant due to inadequate PBMCs count (for many of the maternal samples) and resource constraints. Furthermore, in the current study we could not consider vitamin B12 deficiency or pernicious anemia or gastritis as an exclusion criterion. Future trial considering all these conditions as exclusion criteria will give more precise and concrete evidence of beneficial effect of oral vitamin B12 supplementation during pregnancy on maternal and infant oxidative stress response as well as validate our findings.

## Conclusions

Our findings suggest that maternal B12 supplementation during pregnancy through postpartum can modulate 8-OH-dG and balance cytokine expression to confer protection against immune response-induced oxidative stress. This study indicates a direction for future studies to explore the therapeutic potential of B12 against inflammatory oxidative stress when provided at higher doses such as used in this study. Both milk B12 and maternal plasma B12 concentrations at 3 months were associated with the infants’ plasma B12 concentration. Further analysis of larger cohorts at multiple time points is warranted to confirm this association in other populations. Whether a low concentration of vitamin B12 in milk correlates with the B12 status of infants is a particularly needed avenue of investigation. Is there a threshold below which milk B12 influences infant B12 status? Such research will be critical to improve our understanding of the mode of transfer of B12 from milk to infants.

### Supplementary Information

**Additional file 1:** **Supplementary Table 1.** Plasma concentrations of vitamin B12 (pmol/L), MMA (nmol/L), and tHcy (µmol/L) at 3-months postpartum. **Supplementary Table 2.** Associations between breast milk B-12 concentrations (at 3 months postpartum) and maternal and infant’s plasma B12 biomarker status during pregnancy and at 3 months postpartum.

## Data Availability

Additional supporting material will be available upon request to the corresponding author.
