# Supplementary material for: Effects of vitamin B12 supplementation on oxidative stress markers and pro-inflammatory cytokines during pregnancy and postpartum among Bangladeshi mother–child pairs
Source: BMC Nutr. 2024 Jan 3;10:3. doi: 10.1186/s40795-023-00785-y (PMC10765711; doi:10.1186/s40795-023-00785-y)
Supplement: Supplementary file 1 — Additional file 1: Supplementary Table 1. Plasma concentrations of vitamin B12 (pmol/L), MMA (nmol/L), and tHcy (µmol/L) at 3-months postpartum. Supplementary Table 2. Associations between breast milk B-12 concentrations (at 3 months postpartum) and maternal and infant’s plasma B12 biomarker status during pregnancy and at 3 months postpartum. [file 40795_2023_785_MOESM1_ESM.docx]

**Effects of vitamin B12 Supplementation on Oxidative Stress markers and Pro-Inflammatory Cytokines during Pregnancy and Postpartum among Bangladeshi Mother-child pairs**

Towfida Jahan Siddiqua^1^*, Evana Akhtar^1^, Md. Ahsanul Haq ^1^, Saterah Shahab-Ferdows ^2^, Daniela Hampel^2,3^, Sharmin Islam^1^, Tahmeed Ahmed^1^, Lindsay H Allen^2^, Rubhana Raqib^1^

^1^International Centre for Diarrheal Disease Research, Bangladesh; ^2^USDA ARS Western Human Nutrition Research Center, Davis, CA, USA; ^3^Department of Nutrition, University of California, Davis, CA, USA

^*^**Correspondence:** Dr Towfida J Siddiqua, Director, JiVitA Project (JHUB), Faijan Vila, House # 3, Road # 1, Nasirabad, Keranipara, Rangpur-5400, Phone # +880-1713-202558 (Office). E‐mail: [towfida.jivita@gmail.com](mailto:towfida.jivita@gmail.com)

**Keywords:** Vitamin B12; lactation; human milk; oxidative stress; cytokines

**Supplementary Table 1.** Plasma concentrations of vitamin B12 (pmol/L), MMA (nmol/L), and tHcy (µmol/L) at 3-months postpartum

|  | **Placebo (*n* = 36)** | | **Vit B-12 (*n* = 33)** | | ***P*-value** | |  |
| --- | --- | --- | --- | --- | --- | --- | --- |
| Mother at 3 months |  | |  | |  | |  |
| Hb (g/L) | 14.0±4.4 | | 16.0±3.7 | | 0.067 | |  |
| Folate, nmol/L | 7.2±4.0 | | 6.52±3.07 | | 0.451 | |  |
| B12, pmol/L | 261.7±86.6 | | 434±201 | | <0.001 | |  |
| tHcy, µmol/L | 10.4±3.1 | | 10.07±2.44 | | 0.644 | |  |
| MMA, nmol/L | 397±258 | | 210.7±94.6 | | 0.0002 | |  |
| Child at 3 months |  | |  | |  | |  |
| Hb (g/L) | 10.9±3.2 | | 11.3±3.4 | | 0.655 | |  |
| Folate, nmol/L | 15.5±4.9 | | 14.0±4.2 | | 0.207 | |  |
| B12, pmol/L | 214.6±86.7 | | 332±129 | | <0.001 | |  |
| tHcy, µmol/L | | 13.5±4.1 | | 9.3±2.6 | | <0.001 | |
| MMA, nmol/L | 469.4±309.3 | | 416±530 | | 0.618 | |  |

Data are presented as mean ± SD

Independent sample *t*-tests were used to estimate *p*-values.

Note: Hb: Hemoglobin; tHcy: total homocysteine; MMA: Methylmalonic Acid

**Supplementary Table 2.** Associations between breast milk B-12 concentrations (at 3 months postpartum) and maternal and infant’s plasma B12 biomarker status during pregnancy and at 3 months postpartum

| **Breast milk B-12** | Overall (*n* = 65) | | B12 group (*n* = 32) | | Placebo group (*n* = 33) | |
| --- | --- | --- | --- | --- | --- | --- |
| **at 3 months** | β-(95% CI) | *P*-value | β-(95% CI) | *P*-value | β-(95% CI) | *P*-value |
| **Mother, baseline** |  |  |  |  |  |  |
| Plasma B12 | **628 (209, 1047)** | **0.004** | 627(-70, 1323) | 0.076 | **528 (25, 1031)** | **0.040** |
| Plasma MMA | -164(-477, 150) | 0.300 | -28 (-668, 612) | 0.930 | -301(-617, 14) | 0.061 |
| Plasma tHcy | 40 (-553, 634) | 0.892 | 704 (-288, 1695) | 0.156 | -**757 (-1407, -107)** | **0.024** |
| **Mother, 3 months postpartum** | | |  |  |  |  |
| Plasma B12 | **512 (158, 872)** | **0.005** | **841(336, 1345)** | **0.002** | -133 (-841, 575) | 0.703 |
| Plasma MMA | -202 (-501, 96) | 0.180 | -566 (-1661, 530) | 0.297 | -109 (-431, 212) | 0.493 |
| Plasma tHcy | -205 (-867, 457) | 0.538 | -868 (-2097, 360) | 0.158 | 165 (-580, 910) | 0.654 |
| **Infant, 3 months** |  |  |  |  |  |  |
| Plasma B12 | 277 (132, 423) | **<0.001** | 225 (-18, 469) | 0.068 | 138 (-116, 393) | 0.275 |
| Plasma MMA | -161 (-704, 382) | 0.554 | 16 (-1180, 1211) | 0.978 | -364 (-1149, 421) | 0.350 |
| Plasma tHcy | **-7.63 (-12.40, -2.86)** | **0.002** | -5.43 (-10.06, -0.80) | 0.023 | 2.04 (-9.73, 13.80) | 0.726 |
| **Maternal plasma B12, 3 months postpartum** | | | |  |  |  |
| Infant plasma B12 | **277.5 (132, 423)** | **<0.001** | 225.5 (-18.0, 469) | 0.068 | 138.5 (-116, 393) | 0.275 |
| Infant plasma MMA | 182.9 (-231, 597) | 0.380 | -788.3 (-2321, 745) | 0.298 | 274.8 (-122, 671) | 0.167 |
| Infant plasma tHcy | 1.23 (-7.46, 9.93) | 0.778 | -0.28 (-9.91, 9.35) | 0.953 | -0.42 (-12.54, 11.69) | 0.943 |

A multivariate regression model adjusted for the mother’s age, mode of delivery and the sex of the child, was used to estimate the *p*-values.

Note: B12: Vitamin B12; tHcy: total homocysteine; MMA: Methylmalonic Acid
